# Transmission of new CRF07_BC Strains with 7 amino acid deletion in Gag p6

**DOI:** 10.1186/1743-422X-8-60

**Published:** 2011-02-10

**Authors:** Meng Zhefeng, Hu Huiliang, Qiu Chao, Sun Jun, Lu Jianxin, Zhang Xiaoyan, Xu Jianqing

**Affiliations:** 1 Shanghai Public Health Clinical Center, Institutes of Biomedical Sciences, Fudan University, 2901 Caolang Road, Research Center, Jinshan District, Shanghai, China, 201508; 2 Zhejiang Provincial Key Laboratory of Medical Genetics, School of Laboratory Medicine of Wenzhou Medical College, University-town, Wenzhou, Zhejiang Province, China, 325035; 3 Key Laboratory of Infectious Disease Prevention and Control, China CDC, Beijing, China

## Abstract

A 7 amino acid deletion in Gag p6 (P6delta7) emerged in Chinese prevalent HIV-1 strain CRF07_BC from different epidemic regions. It is important to determine whether this mutation could be transmitted and spread. In this study, HIV-1 Gag sequences from 5 different epidemic regions in China were collected to trace the transmission linkage and to analyze genetic evolution of P6delta7 strains. The sequence analysis demonstrated that P6delta7 is a CRF07_BC specific deletion, different P6delta7 strains could be originated from different parental CRF07_BC recombinants in different epidemic regions, and the transmission of P6delta7 strain has occurred in IDU populations. This is for the first time to identify the transmission linkage for P6delta7 strains and serves as a wake-up call for further monitoring in the future; In addition, P6delta7 deletion may represent an evolutionary feature which might exert influence on the fitness of CRF07_BC strain.

## Findings

Several studies reported that mutations in HIV-1 Gag p6 played no role or only marginal role in the infection and the replication of HIV-1 *in vitro*[1-3]. However, it remains unknown whether those mutations in p6 could exert influences on HIV-1 during natural infection and thereby cause the transmission of those mutated strains. Recently, a new Gag p6 mutation pattern, 7 amino acid (aa) deletion in the central region of p6 domain (PIDKELY at amino acid 30-36, designated as P6Δ7), emerged in CRF07_BC infected individuals in Xinjiang Uygur Autonomous Region of China and has progressively affected nearly 30% CRF07_BC infected population [4]. In addition, P6Δ7 deletion was also identified in CRF07_BC strains circulating in other epidemic sites in China mainland and even in Taiwan region [5-9].

Interestingly, though early cross-sectional observation by Song et al did not observe significant influences of P6Δ7 mutation on biological properties of CRF07_BC, prolonged longitudinal follow-up revealed that P6Δ7 deletion might result in the improvement of CRF07_BC fitness *in vivo*. First, after P6Δ7 mutation occurs *in vivo*, the mutated strain will subsequently replace its parental strain and become the predominant strain; In contrast, the reversion from P6Δ7 strain to non-P6Δ7 strain has never been observed so far; Second, viral loads in P6Δ7 CRF07_BC infected subjects will be more rapidly increased than that in non-P6Δ7 strain infected individuals (Additional file 1, unpublished data). These data suggested that P6Δ7 deletion may have important implications for CRF07_BC prevalence.

Since CRF07_BC strain is one of the most prevalent HIV-1 strains in China [4-9], the appearance of P6Δ7 CRF07_BC strains in different epidemic regions raised several important concerns. First, does P6Δ7 represent a feature only in CRF07_BC or also in other BC recombinant forms? Second, is P6Δ7 strain able to transmit and spread in population? To answer those questions above, we analyzed Chinese-derived Gag full-length sequences collected from all publicly accessible databases and traced the transmission linkage among CRF07_BC P6Δ7 strains which were derived from 4 different provinces and 1 region in China. Interestingly, P6Δ7 was proved to be a CRF07_BC specific mutation and could be originated from different CRF07_BC strains; Furthermore, the mutant strains of P6Δ7 could be transmitted in population in epidemic regions and thereby may cause a new prevalence in the future.

**Figure 1:**
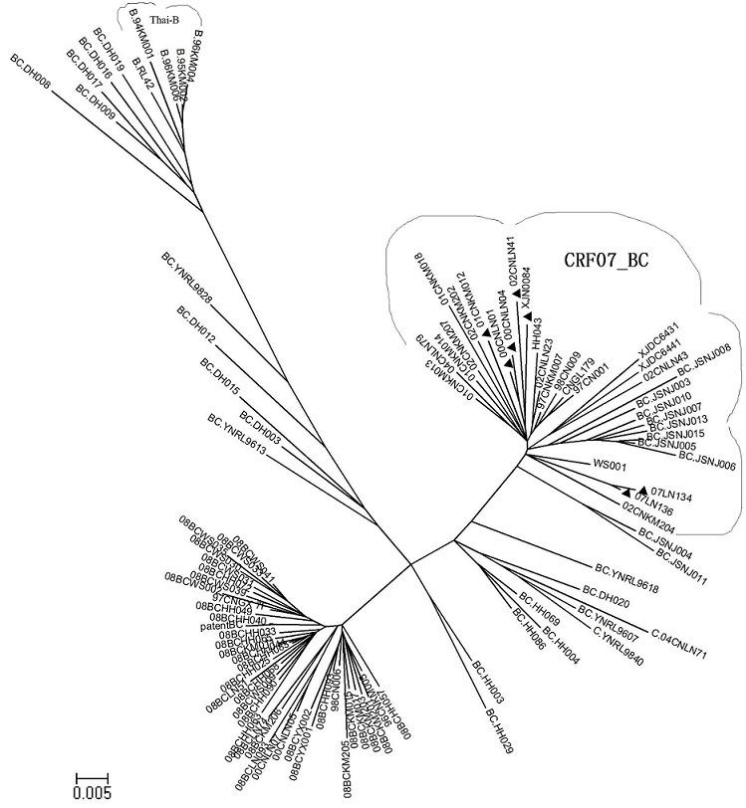
**Phylogenetic tree constructed with 98 Chinese-derived HIV-1 BC recombinant *gag *sequences from HIV database**. The neighbor-joining tree was constructed by Mega4.0. CRF07_BC strains forms an independent cluster and P6Δ7 strains were all clustered within CRF07_BC strains. ▲represents P6Δ7 strains.

98 Chinese derived HIV-1 Gag sequences from HIV database http://www.hiv.lanl.gov, including 27 CRF07_BC, 33 CRF08_BC, 31 BC URFs (unique recombinant forms), 2 India-C and 5 Thai-B, were collected and analyzed by neighbor-joining phylogenetic tree (Figure 1). Interestingly, though CRF07_BC, CRF08_BC and other BC recombinants were derived from the same parental strains (Thai-B and India-C), P6Δ7 was only identified in CRF07_BC (Figure 1). In total, 27 full-length CRF07_BC Gag sequences were available in HIV database, these sequences were derived from 4 provinces and 1 region, including 10 from Yunnan, 8 from Liaoning, 6 from Xinjiang, 2 from Guangxi provinces and 1 from Taiwan region. Among 27 published sequences, 8 (30%) contain P6Δ7 mutation, which is in accordance with previous report in population level that this deletion appeared in 30% CRF07_BC recombinant infected subjects from Xinjiang province [4].

**Figure 2:**
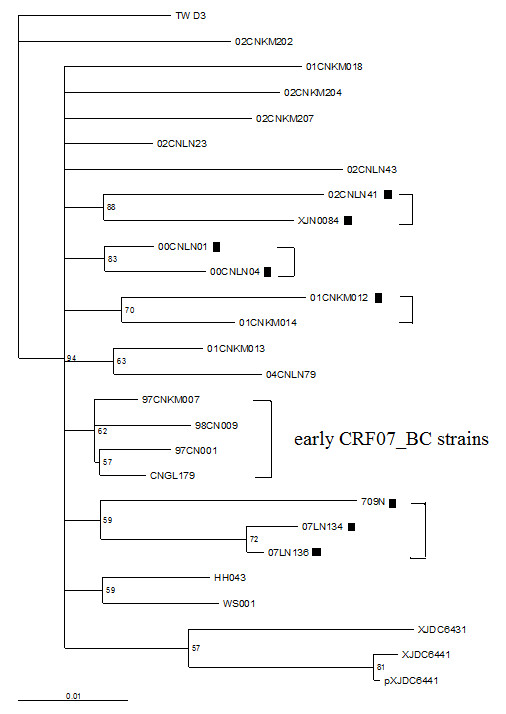
**Maximum-likelihood tree of 27 CRF07_BC Gag sequences derived from HIV database**. 01CNKM012 and 01CNKM014, 00CNLN01 and 00CNLN04, 02CNLN41 and 709, 07LN134 and 07LN136 were paired respectively by more than 70% bootstrap probability and identified as a transmission linkage. ▮ represents P6Δ7 CRF07_BC strains.

To analyze 27 CRF07_BC sequences, the general time-reversible model with a proportion of invariant sites and gamma distribution (GTR+γ+R) was selected as the most appropriate analysis model by Modeltest software [10] and subsequently phylogenetic trees were reconstructed by using a maximum likelihood (ML) heuristic search in PAUPv4.0b10 [11]. Two P6Δ7 sequences (07LN134 and 07LN136) were clustered in one branch with high bootstrap (Figure 2), and epidemiological study demonstrated that they were derived from an IDU couple in Liaoning province, China. Another cluster of P6Δ7 sequences was observed for 00CNLN01 and 00CNLN04, and these two sequences were derived from two IDUs who had shared injection needles during their intravenous drug usage. For these two clusters, both Kishino-Hasegawa test and Shimodaira-Hasegawa test [12,13] showed that the transmission linkage was accepted by p value > 0.95.

**Figure 3:**
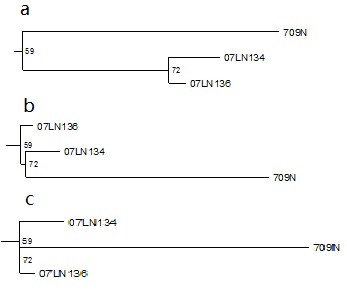
**Tree topologies compatible with the hypothetical transmission linkage (709N-07LN134 and 07LN136)**. All three evolutionary relationships match the transmission linkage depending on the scenario of ancestral diversity and lineage sorting.

**Figure 4:**
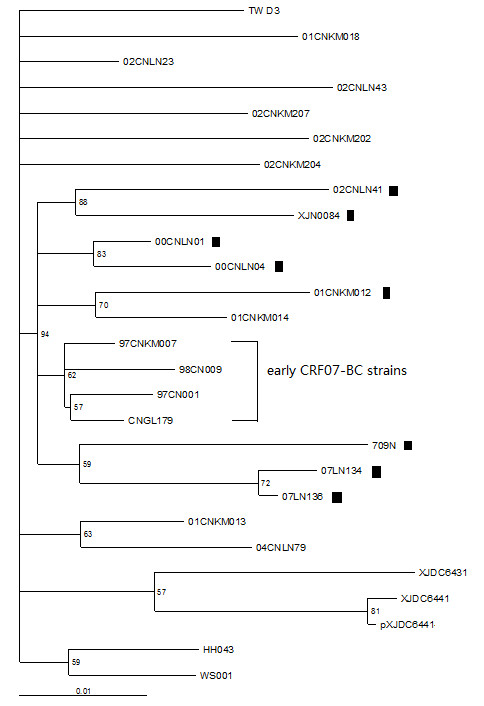
**Reconstructed Maximum-likelihood tree against Figure 2**. P6Δ7 CRF07_BC strains clustered with early CRF07_BC isolate sequences was not rejected by Kishino-Hasegawa test and Shimodaira-Hasegawa test (0.05 < p < 0.95). ▮ represents P6Δ7 CRF07_BC strains.

**Figure 5:**
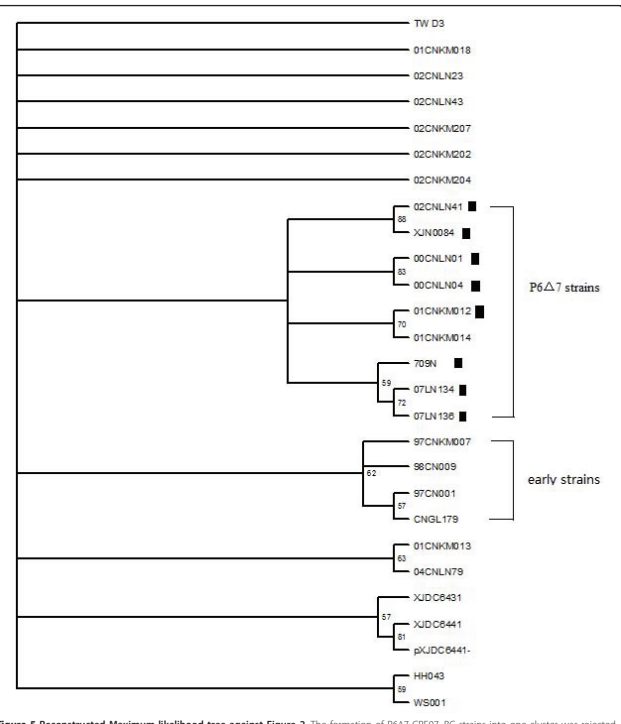
**Reconstructed Maximum-likelihood tree against Figure 2**. The formation of P6Δ7 CRF07_BC strains into one cluster was rejected by Kishino-Hasegawa test and Shimodaira-Hasegawa test (p < 0.05). ▮ represents P6Δ7 CRF07_BC strains.

To test the compatibility of the reconstructed evolutionary relationship with the proposed transmission linkage, the ML trees with different tree topologies were compared by using the Kishino-Hasegawa test and the Shimodaira-Hasegawa test in Consel [12,13]. As shown in Figure 3, all tree topologies are compatible with the hypothetical transmission from 709N to 07LN134 and 07LN136. However, in this case, the original donor may be varied in the tree depending on possible transmission. To test compatibility under this circumstance, we tested any of these topologies against ML tree. The reconstructed maximum-likelihood tree (Figure 4) showed that all P6Δ7 strains were clustered together with early CRF07_BC strains (97CNKM007, 97CN001, CNGL179, and 98CN009), which were not rejected by the Kishino-Hasegawa test and the Shimodaira-Hasegawa test with p value in the range of 0.05~0.95, indicating those P6Δ7 strains could be originated from early non-deletion BC recombinant ancestor strain. However, the possibility that all P6Δ7 strains form one cluster (Figure 5) was strongly rejected by the Kishino-Hasegawa test and the Shimodaira-Hasegawa test (p = 0.013), suggesting those P6Δ7 strains are not derived from the same deletion ancestor strain. Furthermore, two important observations should be noticed. First, as the earliest identified CRF07_BC P6Δ7 isolates, 00CNLN01 and 00CNLN04 had no obvious transmission linkage with other P6Δ7 isolates even in the same epidemic region (Liaoning province); Second, Yunnan province was considered as BC recombinant originated region [6,8,9], however, P6Δ7 isolate (01CNKM012) from Kunming city in Yunnan province was not clustered with other P6Δ7 isolates, instead, this strain clustered with non-P6Δ7 Kunming isolate 02CNKM014. Overall, these data further supported that different P6Δ7 strains could be independently originated and the transmission of P6Δ7 strains only occurred among IDUs who are closely related and thereby could be defined at the very early phase.

**Figure 6:**
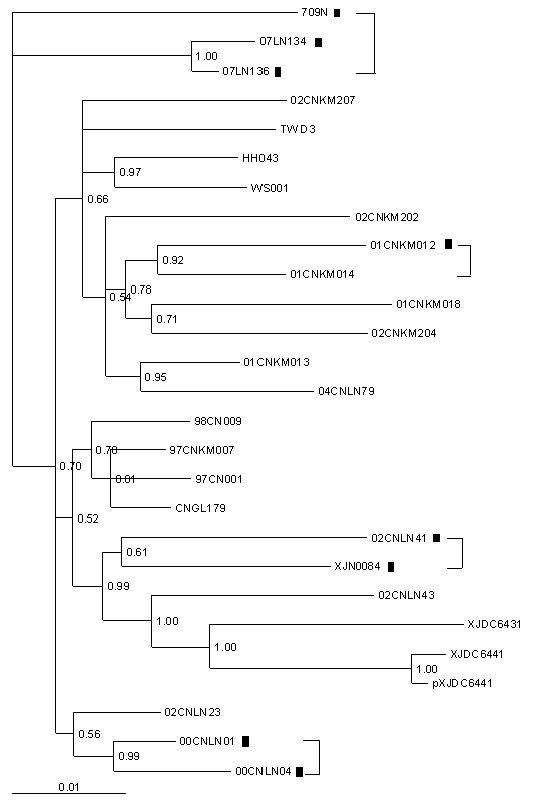
**Maximum-likelihood tree of 27 CRF07_BC Gag sequences constructed by Bayes 3.1**. 01CNKM012 and 01CNKM014, 00CNLN01 and 00CNLN04, 02CNLN41 and 709, 07LN134 and 07LN136 are paired respectively by more than 90% bootstrap probability. ▮ represent represents P6Δ7 CRF07_BC strains.

To further confirm the transmission linkage, Bayesian phylogenetic inference was also performed by employing Markov chain Monte Carlo (MCMC) sampling approach in GTR+γ+R model, as implemented in MrBayes 3.1[11,14]. The MCMC search was run for 10^7 ^generations with trees sampled every 1000th generation. Burn-in was set at 50% and a posterior consensus tree was generated from 25,000 trees sampled. The posterior probability of nodes on the consensus tree was used as phylogenetic support for clusters. Based on previously reports [11,14], significant linkages were considered as those having bootstrap values > 90% and genetic distances < 0.03 nt substitutions per site for *gag *sequences. As expected, the Maximum-likelihood tree constructed by Bayes method also confirmed the transmission linkage of 07LN134 and 07LN136, 00CNLN01 and 00CNLN04, 01CNKM012 and 01CNKM014 (Figure 6). Interestingly, although the pairs were supported by high bootstrap for sequence clusters of 02CNLN41 and XJN0084, 709N and 07LN134/07LN136, the transmission linkage was not supported by both the Kishino-Hasegawa test and the Shimodaira-Hasegawa test with p value below 0.95, and those sequences were actually derived from IDUs who reside in different provinces by thousands miles apart. These data indicated that approaches employed here to test the transmission linkage are reliable and CRF07_BC P6Δ7 strains could be transmitted among IDUs.

**Figure 7:**
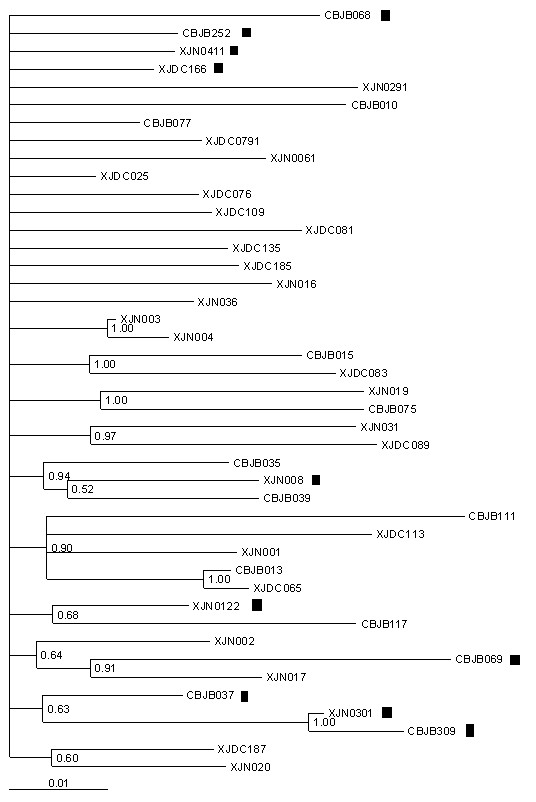
**Maximum-likelihood tree of 43 CRF07_BC Gag sequences from Xinjiang Uygur Autonomous Region of China was constructed by Bayes3.1**. XJN0301 and CBJB309, XJN017 and CBJB069 are paired respectively by more than 90% bootstrap probability. ▮ represent represents P6Δ7 CRF07_BC strains.

The same analysis was performed for additional 43 CRF07_BC *gag *sequences which were collected from IDU subjects in Urumqi city in Xinjiang Uygur Autonomous Region, China, as described previously [4]. Maximum-likelihood tree was constructed by MrBayes 3.1 (Figure 7). Among 43 sequences, 10 sequences were identified containing P6Δ7 mutations. Similar to the results from the analysis above, the transmission linkages between P6Δ7 isolates (XJN0301 and CBJB309), or between P6Δ7 isolates and non-P6Δ7 isolates (CBJB069 and XJN017) were supported by both Kishino-Hasegawa test and Shimodaira-Hasegawa test (p > 0.95). These data confirmed the observations above that P6Δ7 strains could be originated independently and the transmission of P6Δ7 strains did occur.

It remains controversial whether mutations in p6 could exert influence on the infection and the replication of HIV-1. Pikora CA et al and Bleiber G et al showed that deletion up to 18 aa (S14-I31) in p6 only had minor effects on the infectivity of HIV *in vitro *[2,3]; In contrast, Lazert C et al observed that 4 aa deletion in the central region of p6 (Δ25SQKQ28) increased its association with ALIX which serves as a chaperone protein to facilitate the viral assembling and budding process [15]. Different from the artificial deletions in p6 as described above, P6Δ7 deletion in CRF07_BC strains is naturally occurred as a unique mutation pattern in a specific subtype, which suggested that this mutation may have important implication for CRF07_BC; Indeed, our longitudinal follow-up observed that P6Δ7 deletion resulted in the rapid increase in viral loads (Additional file1). In addition, this deletion was not observed in CRF08_BC. As p6 Gag in CRF07_BC is derived from B clade whereas p6 Gag in CRF08_BC is from C clade, and the backbone of CRF_BC is derived from C clade, P6Δ7 deletion may represent a new adaption of B clade derived p6 Gag to C clade derived backbone.

CRF07_BC is the most prevalent Chinese strains and accounts for nearly half of HIV-1 infection across the nation [4-9], suggesting that this recombinant has been highly adapted in Chinese population and any mutations in this strain needs to be closely monitored. P6Δ7 mutation was observed in a fraction of CRF07_BC infected subjects [4], the next important question for public health is whether this mutation could be transmitted and spread. Our sequence analysis demonstrated that the transmission of P6Δ7 strains did occur in populations. This is for the first time to establish the transmission linkage for P6Δ7 strains; Importantly, the transmission has occurred in different epidemic regions. Therefore, these data serve as a wake-up call for our authority. Since the transmission of P6Δ7 strains was only observed between epidemiologically closely related IDUs, it is speculated that this is the initial phase for the transmission of P6Δ7 strains. In addition, our data also established that P6Δ7 CRF07_BC could be originated from different parental strains and thereby had versatile original ancestors in evolution.

Both the independent occurrence of the P6Δ7 in different CRF07_BC infected individuals and the transmission of P6Δ7 strain among IDUs suggested that this deletion may have important implications. As known, HIV-1 Gag p6 protein play a critical role in viral particle budding by interaction with host factor Tsg101 and ALIX [15-18], there may exist active mechanisms for host cells to interrupt this process and thereby block the viral budding. Therefore, it is rationalized that P6Δ7 may represent a new recombinant form escaping from anti-p6 based budding mechanism. In this regards, it will be important to address how P6Δ7 will influence the engagement of p6 into the budding process.

## List of abbreviations

HIV: Human Immunodeficiency Virus; CRF: Circulating recombinant form; IDU: Injection Drug User;

## Competing interests

The authors declare that they have no competing interests.

## Authors' contributions

MZ conceived the study, carried out the molecular genetic studies, participated in the sequence alignment and drafted the manuscript. HH and QC and SJ participated in the sequence alignment and participated in the design of the study and performed the probability testing of phylogenetic tree. LJ coordinated the study, participated in the experimental design and helped to draft of the manuscript. XJ and ZX proposed the concept of the study, designed the study, formulated the major conclusion and revised this manuscript, and all authors read and approved the final manuscript.

## Supplementary Material

Additional file 1 **Comparison of viral load and viral load change between non-deletion and P6Δ7 CRF07_BC strains infected patients**. 11 non-deletion strains patients and 7 P6Δ7 CRF07_BC strains patients was consecutively follow-up for 2-3 years. No significance difference was detected in the initial viral load(infection time < 6 months) of these two groups, whereas viral load of P6Δ 7 was higher and increases more rapidly than that of non-deletion in last follow-up (P < 0.05). Click here for file
