# Supplementary figures and images for: Transmission of new CRF07_BC Strains with 7 amino acid deletion in Gag p6
Source: Virol J. 2011 Feb 10;8:60. doi: 10.1186/1743-422X-8-60 (PMC3048562; doi:10.1186/1743-422X-8-60)

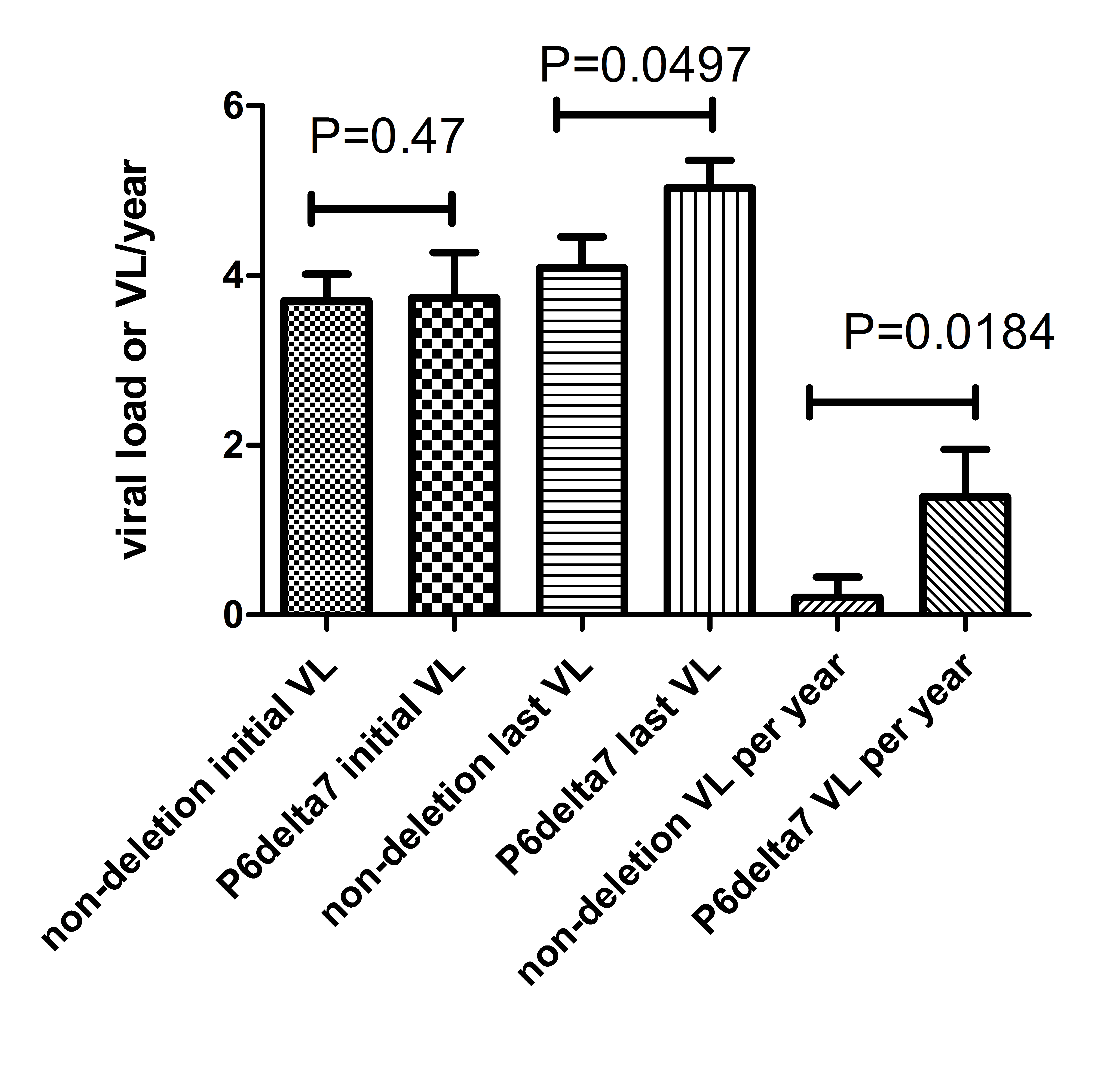

Supplement: Additional file 1 — Comparison of viral load and viral load change between non-deletion and P6Δ7 CRF07_BC strains infected patients. 11 non-deletion strains patients and 7 P6Δ7 CRF07_BC strains patients was consecutively follow-up for 2-3 years. No significance difference was detected in the initial viral load(infection time < 6 months) of these two groups, whereas viral load of P6Δ 7 was higher and increases more rapidly than that of non-deletion in last follow-up (P < 0.05). [file 1743-422X-8-60-S1.JPEG]
